# Wrinkling and Strengthening Behaviors in the Two-Layer-Sheet Hot-Forming–Quenching Integrated Process for an Al–Cu–Mg-Alloy Thin-Walled Curved-Surface Shell

**DOI:** 10.3390/ma16134766

**Published:** 2023-07-01

**Authors:** Xiaobo Fan, Baoshan Sun, Wenliang Qu, Xianshuo Chen, Xugang Wang

**Affiliations:** 1 School of Mechanical Engineering, Dalian University of Technology, Dalian 116000, China; 2 School of Materials Science and Engineering, Harbin Institute of Technology, Harbin 150000, Chinawxg6310@126.com (X.W.)

**Keywords:** aluminum alloy, two-layer sheet, hot forming-quenching integrated process, wrinkling, strengthening

## Abstract

The thin-walled curved-surface component is an important structural element in aerospace. Wrinkling, springback and thermal distortion occur easily when forming these components. To form thin-walled components with high precision and strength, a two-layer-sheet hot-forming–quenching integrated process was proposed, in which wrinkling is prevented by thickening the upper sheet and springback is reduced by solution and die quenching. Selecting an appropriate upper sheet is crucial to suppress wrinkling and accomplish effective die quenching. The effect of the upper sheet on the wrinkling and strengthening behaviors of an Al–Cu–Mg-alloy melon-petal shell was thus studied in detail. The anti-wrinkle mechanism was analyzed through numerical simulation. The forming quality, including forming precision, deformation uniformity and strength, were further evaluated. The wrinkle gradually decreased with the increasing thickness of the upper sheet, resulting from the depressed compressive stress at the edge of the target sheet. A defect-free specimen with a smooth surface was finally formed when the thickness of the upper sheet reached three times that of the target sheet. The profile deviation was ±0.5 mm. Excellent thickness uniformity in a specimen can be obtained with a maximum thinning rate of 6%. The full strength, ranging from 455 to 466 MPa, can be obtained in all regions of the specimen, indicating that effective strengthening can be accomplished with the two-layer-sheet die quenching. The results indicated that high forming quality and full strength can be obtained in a two-layer-sheet hot-forming–quenching integrated process. This research has great potential for engineering applications using aluminum-alloy curved-surface thin-walled components.

## 1. Introduction

**Figure 1:**
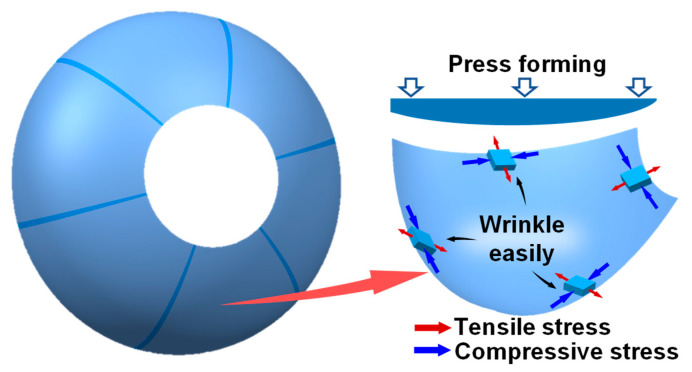
Large-sized dome of rocket fuel tank.

Aluminum alloy has become the primary structural material in the automotive and aerospace industries because of its high strength-to-weight ratio, driven by the increasing need for light structural weight [1]. Currently, the large-sized rocket-fuel-tank dome is normally fabricated by welding together multiple melon petals, which are usually made of high-strength aluminum alloy [2]. Therefore, melon-petal thin shells need high strength and precision to ensure reliable welding [3]. The melon petal is a typical thin-walled curved-surface component with geometric asymmetry. Wrinkling occurs easily in direct press forming for the melon petal, due to the compressive stress and thin walls, as shown in Figure 1. Therefore, the melon petals are usually fabricated by stretch forming (SF) and deep drawing [4,5]. However, splitting occurs easily in these processes, resulting from the poor formability of high-strength aluminum alloy at room temperature. To enhance the formability and the performance of the components, the creep-aging forming (CAF) process has been developed [6]. Poor forming efficiency limits the mass application of the CAF process, and the springback is also very difficult to solve [7,8].

To improve the formability and reduce the springback, the hot-forming–quenching integrated progress (HFQ) has been developed [9,10]. In this process, a solution-treated sheet is quickly transferred to the cold dies, then formed and maintained in the cold dies for high forming precision and efficient strengthening [11]. This process is significantly affected by the solution conditions, the speed of transferring and forming, and the quenching rate [2,12]. A higher forming speed is beneficial for minimal thinning and improving post-form strength in the HFQ process [13,14]. The cold dies have been used to quench to realize rapid quenching and avoid thermal distortion [15]. The forming precision and mechanical properties were studied using two different HFQ process routes, and it was found that U-shaped specimens with high precision and uniform mechanical properties can be formed using HFQ [16]. Although HFQ has been widely used in the automotive and aerospace industries for complex-shaped curved components with superior precision and strength, wrinkling still occurs in the forming process of curved-surface shells with geometric asymmetry like the melon petals of the abovementioned tank. Therefore, new methods need to be developed to inhibit the wrinkling defect in thin-walled melon-petal components.

Multilayer sheet forming has been widely used to suppress wrinkling in the aerospace and automotive industries [17]. Multilayer sheet forming is used to improve the poor formability of sheets at room temperature [18]. The wrinkling behaviors of 5A06 single-layer and two-layer sheets in the hydroforming process have been discussed previously. The results show that the anti-wrinkle ability of the two-layer sheet is significantly enhanced [19,20,21]. In addition, the forming limit can be significantly enhanced in this process [22,23]. Based on numerical simulation and experiment, it was confirmed that the increasing thickness of the upper sheet significantly improved the limit drawing ratio [24]. Springback still occurs in two-layer-sheet press forming at room temperature. Overall, high precision and strength can be obtained by HFQ, but it is difficult to suppress the wrinkling. In contrast, the multilayer sheet forming can efficiently suppress wrinkling, but the springback still occurs. Therefore, a novel two-layer-sheet hot-forming–quenching integrated process was proposed for forming thin-walled curved-surface components like melon petals. The wrinkling can be suppressed by two-layer sheets and the springback can be reduced by die quenching in this novel process. Nevertheless, how to choose a suitable thickness for the upper sheet for controlling the wrinkling and for die quenching is very critical in the two-layer-sheet hot-forming–quenching process.

Therefore, the effect of the upper sheet on wrinkling and strengthening behaviors for an Al–Cu–Mg-alloy melon-petal shell was studied in detail. The anti-wrinkle mechanism was analyzed through numerical simulation of the two-layer-sheet hot-forming–quenching integrated process. The forming quality of the melon petal was further evaluated, including forming precision, deformation uniformity and strength. These results can provide guidance for the precise manufacturing of aluminum-alloy thin-walled curved-surface components, and the novel process provides a new forming route for the manufacture of these components.

## 2. Materials and Procedures

### 2.1. Materials

The as-received material was a heat-treatable Al–Cu–Mg aluminum-alloy rolled sheet with 1 mm in thickness. The chemical composition was Al-4.57Cu-1.49Mg (wt. %). For the Al–Cu–Mg alloy, full strength was obtained at the strengthening level of T4 after the solution heat treatment (490 °C for 30 min) and sufficient natural aging, and the corresponding tensile strength was 450 MPa.

### 2.2. Experimental Procedure

**Figure 2:**
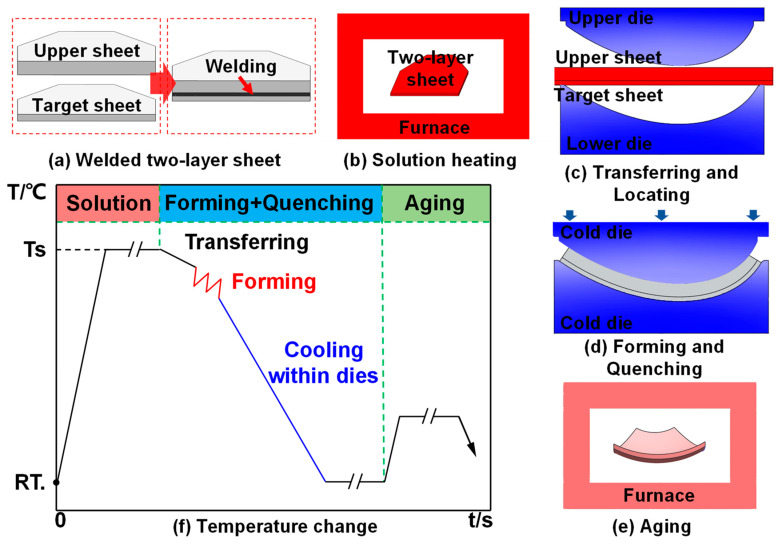
Schematic diagram of two-layer-sheet hot-forming–quenching integrated process.

Figure 2 shows the integrated progress of two-layer-sheet hot-forming–quenching for an aluminum-alloy melon-petal shell. First, the two-layer sheet was obtained by welding together the edges of the target forming sheet and the upper auxiliary sheet. Then, an adequate solution treatment of the two-layer sheet at 490 °C for 30 min was conducted. After that, the post-solution-treatment two-layer sheet was quickly transferred to the lower die within 3 s and formed into components with the target shape within 1 s. The target sheet was placed on the side near the lower die. Meanwhile, the specimen was quenched in the cold die to achieve high forming precision. Finally, a subsequent aging process was carried out to obtain the full strength. Therefore, the novel process with high efficiency can better meet the requirements of the high-frequency launching of rockets compared with other fabricating methods, such as creep-aging forming and additive manufacturing [7,25].

**Figure 3:**
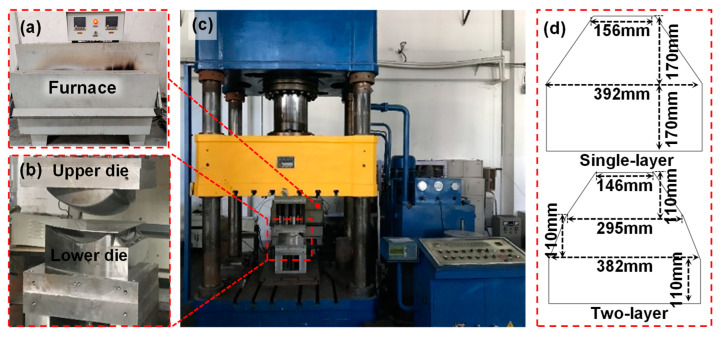
Experimental setup for two-layer-sheet hot-forming–quenching integrated process. (**a**) the air furnace; (**b**) the dies; (**c**) the fast-forming press; (**d**) the size of sheet.

Figure 3 shows the experimental setup for the two-layer-sheet hot-forming–quenching integrated process, mainly including a fast-forming press, cold dies and an air furnace. The die clearance between the upper and lower dies was designed to be the same as the thickness of the two-layer sheet. The volume of the dies is large enough compared with the sheet blank to ensure sufficient cooling capacity. The sheet was solution-heated in an air furnace with the accuracy of ±1.0 °C. The clamping force was about 25 t, provided by the fast-forming press. The size of the designed specimen is shown in Figure 3d. The target forming component was designed as a 1/6 melon-petal shell with a semi-major axis of 333.8 mm and a semi-minor axis of 208.6 mm. The thickness of the target was 1 mm, which contributes to wrinkling easily.

To study the effect of the thickness of the upper auxiliary sheet on the deformation behavior of the target sheet, three typical thicknesses (1 mm, 2 mm and 3 mm) were selected. Accordingly, the clearances between the upper die and lower die were determined to be 2 mm, 3 mm and 4 mm. To evaluate the forming precision, the dimensional deviation was measured by a Powerscan 3D scanner with an accuracy of 0.02 mm. Meanwhile, the deviations before and after removing the edge constraint were compared. The thickness uniformity was reflected by the thickness distribution in different regions of the component. Uniaxial tensile tests in different areas of the formed specimen were implemented to evaluate the strengthening behavior.

### 2.3. Numerical Simulation

**Figure 4:**
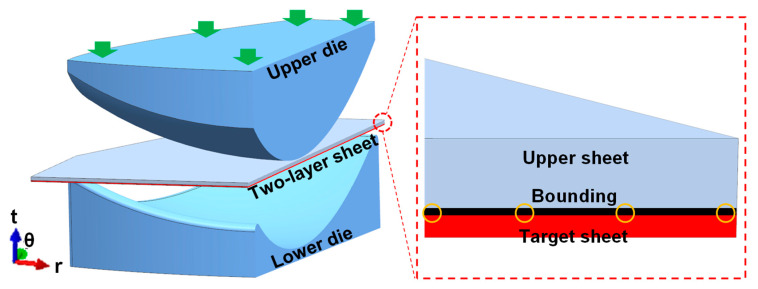
Numerical simulation model.

The anti-wrinkling mechanism of the two-layer-sheet hot-forming–quenching integrated progress was studied by numerical simulation. Figure 4 shows the numerical simulation model, performed by Abaqus 6.14. The upper die and lower die were defined as discrete rigid bodies. The two-layer sheet was defined as an isotropic deformable body meshed by a hexahedral structure with a type of C3D8T. The element of the target sheet was designed as 3.5 mm and five layers were divided in thickness. The elements number of the target sheet was about 25 thousand. The elements structure of the upper sheet was the same as the target forming sheet. The welding constraint between the target sheet and the upper sheet was realized by the bounding of edges. The friction coefficient of the dies–two-layer sheet was set as 0.15. The friction coefficient of the upper sheet–target sheet was particularly set as 0.25 in order to suppress wrinkling more efficiently. The total displacement of the upper die was about 70.0 mm. The effect of the upper sheet’s thickness (1 mm, 2 mm and 3 mm) on the stress distribution and wrinkling behavior of the ellipsoidal shell in the two-layer-sheet hot-forming–quenching integrated process was analyzed.

## 3. Results and Discussion

### 3.1. Wrinkling Behavior

**Figure 5:**
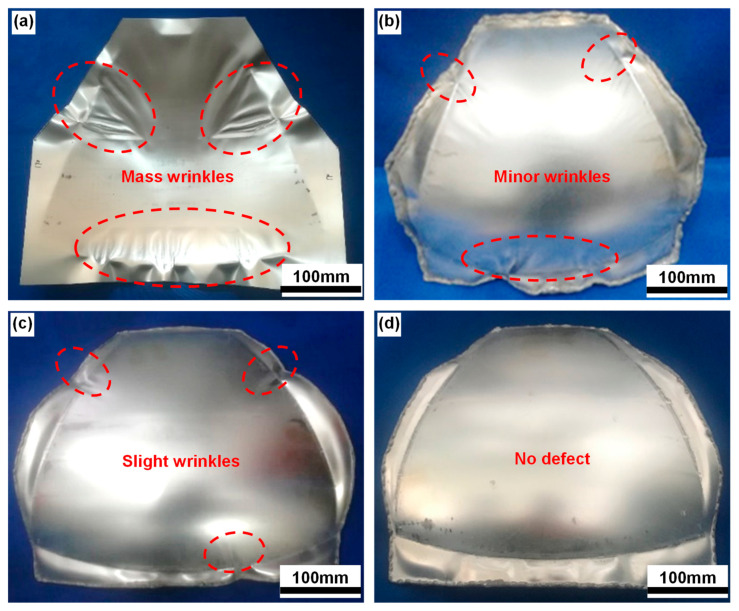
Post-forming specimens using upper sheets with different thickness: (**a**) 0 mm, (**b**) 1 mm, (**c**) 2 mm and (**d**) 3 mm.

Figure 5 shows the post-forming specimens using upper sheets with different thicknesses. Serious wrinkling and folding occurred when the auxiliary sheet was not used (Figure 5a). Wrinkling was gradually suppressed with the increase in the upper sheet’s thickness, as shown in red zone in Figure 5b–d. Wrinkling disappeared when the thickness of the upper auxiliary sheet increased to 3 mm, and a specimen with a smooth surface and no defects could be finally formed. The results indicated that thin-walled curved-surface components can be formed by the two-layer-sheet hot-forming–quenching integrated process.

**Figure 6:**
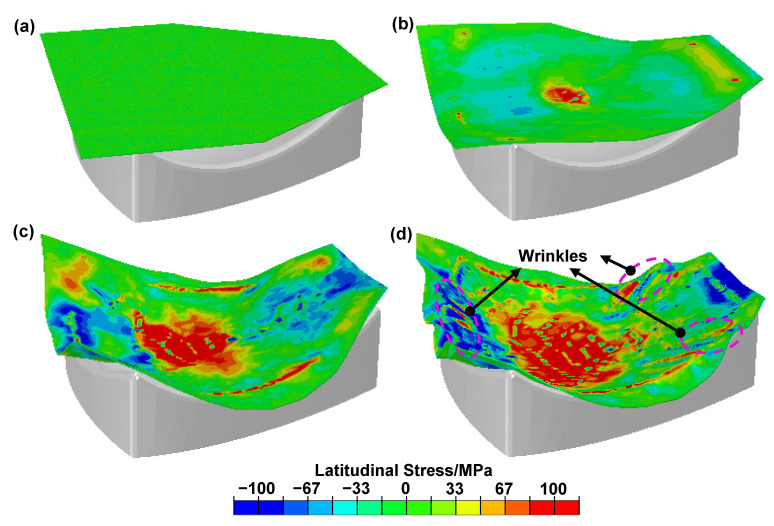
Changes in stress and shape in different deformation stages: (**a**) 0%, (**b**) 40%, (**c**) 80% and (**d**) 100%.

A numerical simulation was carried out to reveal the wrinkling behavior of the sheets in different forming stages. Figure 6 shows the different forming stages of the melon-petal shell. As the upper die moves down, the sheet is first bent, which is manifested by warping on both sides and sinking inside (Figure 6b). At this point, the edge of the sheet is subjected to compressive stress. In the subsequent deformation stage, the large side of the sheet is fed less, due to earlier attachment to the dies and greater friction, and the wrinkling occurs due to the significantly increasing compressive stress (Figure 6c). Finally, the upper and lower dies are completely closed (Figure 6d). Obvious wrinkling exists at the edge of the sheet due to the compressive stress and thin wall, which corresponds to Figure 1.

**Figure 7:**
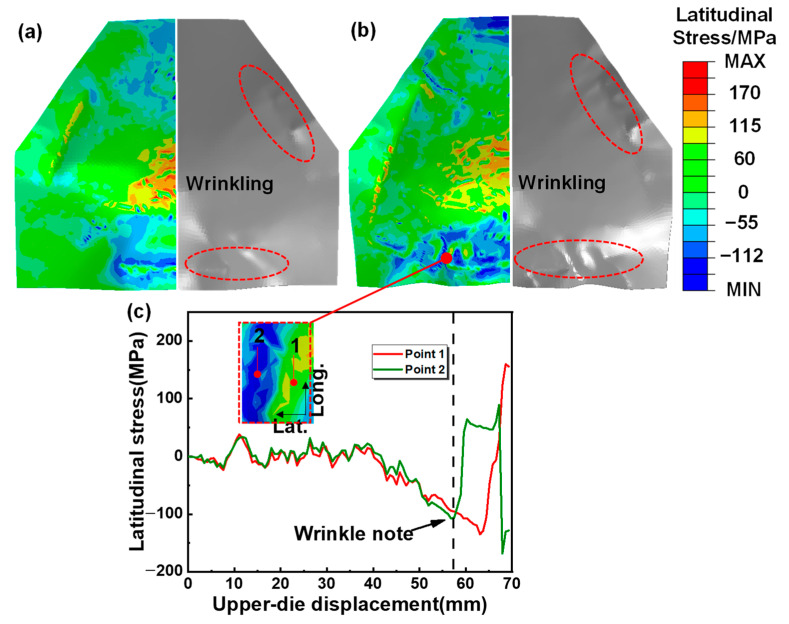
Latitudinal stress of single-layer forming in different deformation stages: (**a**) 80%, (**b**) 100%; and (**c**) latitudinal stress change in wrinkled area in all deformation stages.

**Figure 8:**
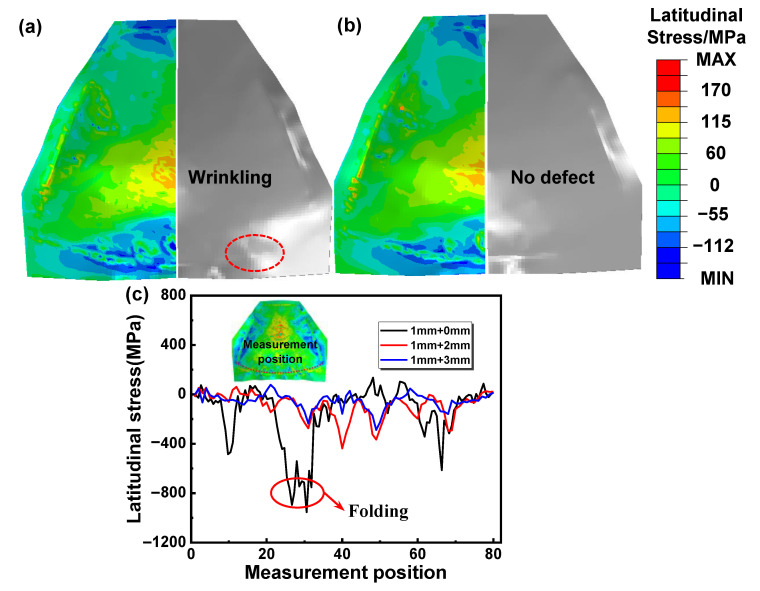
Latitudinal stress of different upper-sheet thicknesses: (**a**) 2 mm, (**b**) 3 mm; and (**c**) latitudinal stress changes of different upper-sheet thicknesses at different measurement positions of larger edge.

Stress distribution was utilized to reveal the anti-wrinkling mechanism. The wrinkling mechanism of single-layer sheets was studied first for comparison. Figure 7 shows the wrinkling behavior and the latitudinal stress distribution inside the sheet for a single-layer sheet, and the red zone shows the wrinkling. Obvious wrinkling can be observed at the edge of the specimens, when the upper die moves down to 80% of the total displacement and the latitudinal compressive stress is up to 100 MPa. The critical wrinkling stress was reduced due to the material softening at elevated temperature. Therefore, in hot forming–quenching for a single-layer sheet, the compressive stress at the edge can easily exceed the critical wrinkling stress, which will lead to wrinkling defects [26]. Figure 8 shows the wrinkling behavior and latitudinal stress distribution for two-layer sheets with upper sheet thicknesses of 2 mm and 3 mm, and the red zone shows the wrinkling. It is obvious that the latitudinal compressive stress gradually decreases with the increase in the upper sheet thickness. Furthermore, the stress in the edge zone tends to be evenly distributed. The wrinkling is completely eliminated when the upper sheet thickness increases to 3mm, which has good consistency with the experimental results. The results show that the compressive stress at the edge of the target sheet can be effectively reduced by the upper auxiliary sheet, thus inhibiting the wrinkling defect. That is to say, two-layer sheet forming is an effective method to prevent the wrinkling of an ellipsoidal shell.

### 3.2. Forming Precision

**Figure 9:**
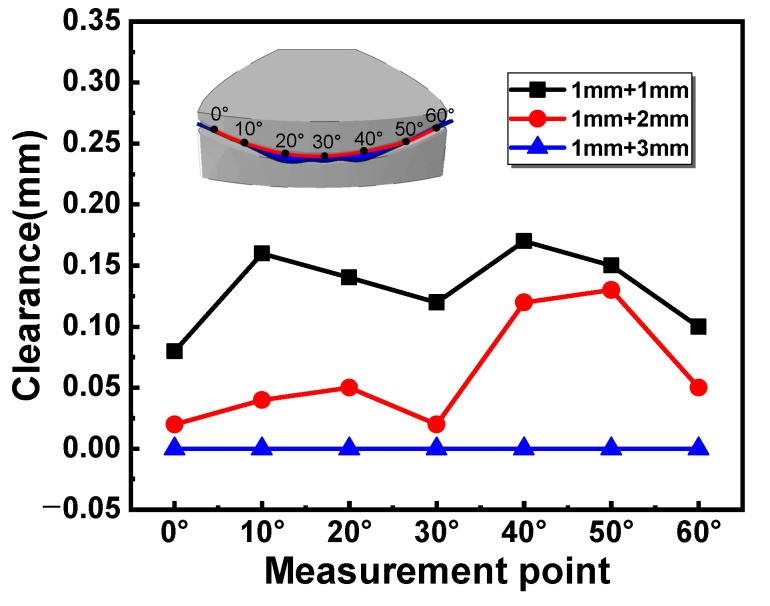
Clearance between the larger edge of the sheets and the upper die after clamping dies.

Figure 9 shows the clearance between the larger edge of the sheets and the upper die after clamping dies. The clearance value was measured every 10 degrees along the larger edge of the two-layer sheet. It is obvious that the clearance decreases with the increase in the upper sheet’s thickness. The larger clearances are located at the larger edge between 10°–20° and 40°–50°, due to the wrinkling in the corresponding position (Figure 5). When the thickness of the upper sheet increases to 3 mm, the clearance is close to 0. The results indicated that the forming precision can be improved by increasing the thickness of the upper auxiliary sheet in the two-layer-sheet hot-forming–quenching integrated process.

**Figure 10:**
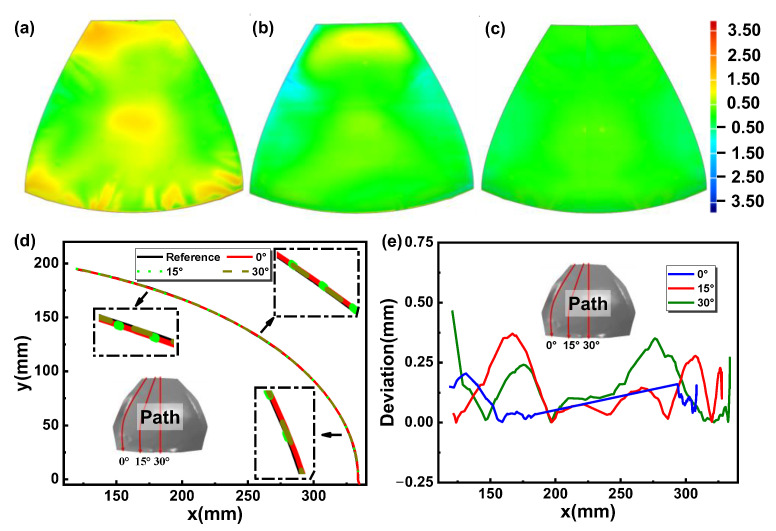
Dimensional deviation under different thicknesses of upper sheets: (**a**) 1 mm, (**b**) 2 mm, (**c**) 3 mm; (**d**) profile comparison between the defect-free specimen and the lower die, and (**e**) profile deviation of the defect-free specimen.

Profile accuracy is the key to determining whether the designed specimen was successfully manufactured. When different post-forming parts are to be welded together, thigh forming precision is needed, which reduces the workload for calibration shape. Figure 10 shows the dimensional deviation between the die cavity and the specimen (Figure 5d), to quantitatively evaluate the forming precision. It can be observed that the dimensional deviation is gradually reduced as the thickness of the upper sheet increases, keeping consistent with the wrinkling trend. The highest forming precision can be obtained under 3 mm thickness of the upper sheet, and the maximum deviation is less than 0.5 mm (Figure 10e). The widely distributed positive deviation is caused by the release of compressive stress. The clearance between dies and specimen is very small after clamping the dies.

**Figure 11:**
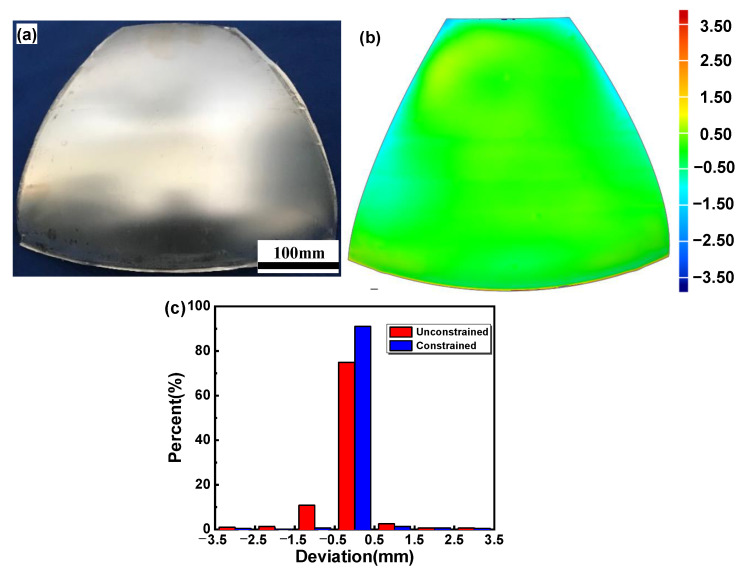
Specimen after removing the welding constraint (**a**), dimensional deviation after removing the welding constraint (**b**) and deviation distribution of target melon petal (**c**).

In addition, the second springback occurs after removing the welding constraint. Figure 11 shows the final forming precision of the specimen (Figure 5d). A larger deviation at the edge appears compared with that before removing the welding constraint. There is no obvious change in the deviation of the central regions. The proportion of deviation distribution between ±0.5 mm decreases from 91.0 % to 75.0 %. The proportion of deviation distribution between ±0.5 mm–1.5 mm increases from 2.0 % to 13.4 %. The results show that the post-forming component using an upper auxiliary sheet with 3 mm thickness was charactered by high forming precision after removing the welding constraint.

Through the above analysis, it was found that the final dimension deviation of the melon petal results from the accumulation of springback after opening the dies and removing the welding constraint. In addition, the springback after removing the welding constraint was dominant. The dimensional deviation was mainly located at the edge of the specimens. Therefore, for the two-layer-sheet hot-forming–quenching process, a process transition edge can be designed and cut off after forming to further improve the final precision of the specimen.

### 3.3. Thickness Distribution

**Figure 12:**
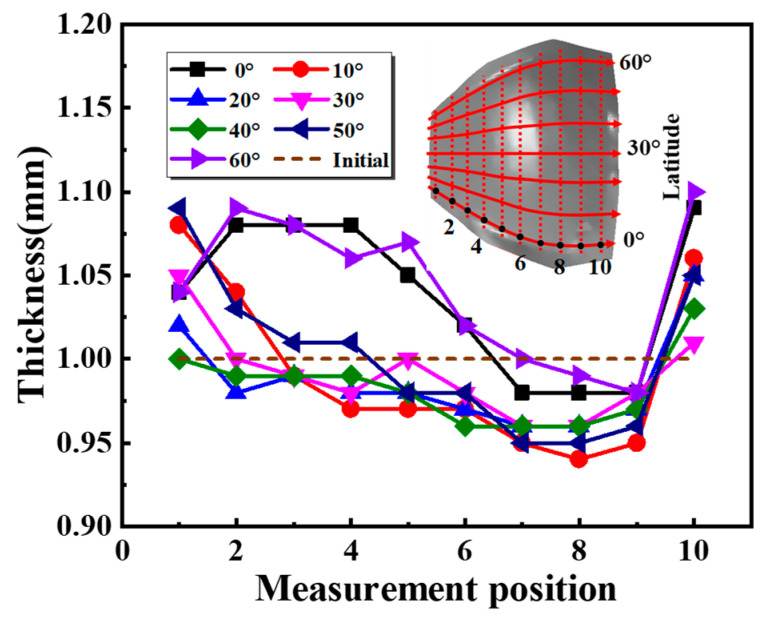
Thickness distribution of defect-free specimen.

Figure 12 shows the thickness distribution of the defect-free specimen (Figure 5d). The maximum thinning rate and the maximum thickening rate of the specimen are 6.0% and 10.0%, respectively, and the maximum deviation is 0.16 mm. Excellent thickness uniformity of the specimen can be obtained with the two-layer-sheet hot-forming–quenching integrated process. In the direction from position 1 to position 10, the thickness distribution trend of different angles is generally consistent. In particular, the thicknesses of the specimen at 0° and 60° are significantly thicker than that at other angles, caused by the compressive stress in areas tending to wrinkle. It is seen that the general trend of thickness distribution along the direction from position 1 to position 10 is thickening on the larger and smaller edges (positions 10 and 1, respectively, as shown in Figure 12), with thinning in the middle. This is because the target sheet is located on the outside of the two-layer sheet, so that the biaxial stress acts on the central area. This is the primary reason for the thinning in the central area. In addition, the larger edge and the smaller edge of the specimen are also thickened. This is also caused by the compressive stress, the same for 0° and 60°. In addition, part of the thickened region around the specimen is located in the process transition edge, which is removed after final forming.

### 3.4. Strength Distribution

**Figure 13:**
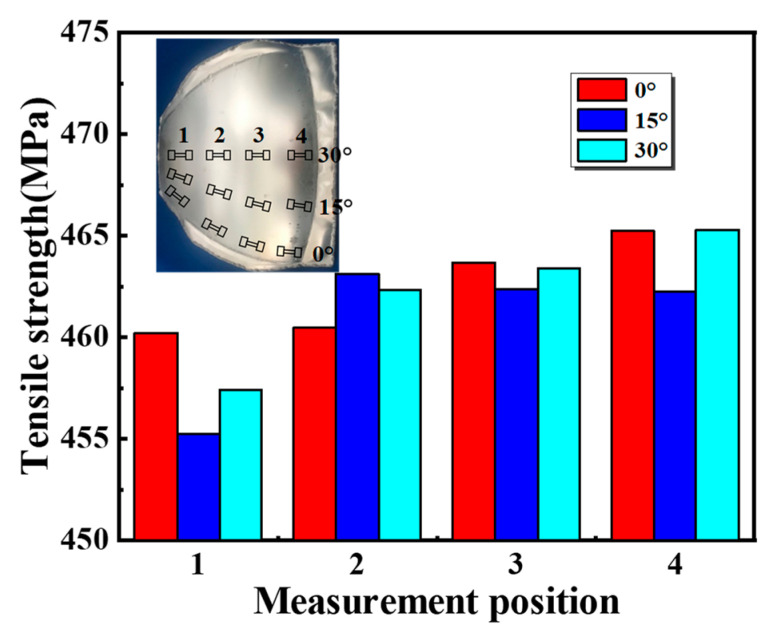
Strength distribution of defect-free specimen in different regions.

Figure 13 shows the tensile strength distribution of the specimen (Figure 5d). The strength of the specimen ranges from 455 MPa to 466 MPa, reaching the full strength of the material [13]. High tensile strength in different regions is obtained. This is because a specimen without wrinkling defects can completely attach to the dies (Figure 9), thus ensuring a high cooling rate and further effective quenching. The above results indicate that the full strength can be obtained by the die cooling in the novel process. In addition, the full strength can be still realized by the method of cooling die quenching, although the quenching thickness is increased by the two-layer sheet. The strengths in the larger edge and the smaller edge have only a small difference, mainly caused by the order of sticking to the dies. The larger edge of the specimen sticked to the dies first, so the strength of the larger edge is relatively higher. However, the maximum difference is only 2.2% of the total strength, showing the great uniformity of strength in different regions. These results suggest that the two-layer-sheet hot-forming–quenching integrated process can ensure effective and uniform quenching.

## 4. Conclusions

In this study, a two-layer-sheet hot-forming–quenching integrated process was proposed to precisely fabricate aluminum-alloy thin-walled melon-petal components. The anti-wrinkle mechanisms, forming precision and strengthening behaviors of an Al–Cu–Mg-alloy curved-surface shell were discussed in detail. The conclusions were as follows:

- The thicker upper sheet is beneficial to suppress wrinkling. When the thickness of the upper sheet increases to three times that of the target sheet, a curved-surface component with a smooth surface and no defects can finally be formed. The upper auxiliary sheet can effectively reduce the compressive stress at the edge of the target forming sheet, thus inhibiting the wrinkling defect.
- A melon petal can be formed with high precision. The profile deviation in the desired region is between −0.5 mm and 0.5 mm. This is due to the fact that a temperature close to the solution temperature and die quenching are utilized to suppress springback and avoid thermal deformation.
- Full strength can be obtained by die quenching in the two-layer-sheet hot-forming–quenching integrated process. The tensile strength in all regions ranges from 455 MPa to 466 MPa, reaching the full strength of the used material and ensuring uniform quenching.
- Thin-walled curved-surface components such as melon petals with high forming quality and full strength can be formed by this two-layer-sheet hot-forming–quenching integrated process, which has great potential for fabricating aluminum-alloy thin-walled curved-surface components.

## Data Availability

The data used to support the findings of this study are available from the corresponding author upon request.
